# Diversity of Gracilariaceae (Gracilariales, Rhodophyta) Across Distinct Ecosystems in Zhanjiang, China: A Foundation for Screening Potential Cultivable Species in Southern China

**DOI:** 10.1002/ece3.71748

**Published:** 2025-07-09

**Authors:** Zhaojun Zeng, Enyi Xie, Huaqiang Tan, Xuefeng Wang, Wencheng Yang, Nenghui Li, Qun Lai, Kun Lin, Manning Lei, Xinlu Wu, Jianjun Cui

**Affiliations:** ^1^ Guangdong Provincial Key Laboratory of Aquatic Animal Disease Control and Healthy Culture, Key Laboratory of Marine Ecology and Aquaculture Environment of Zhanjiang Fishery College of Guangdong Ocean University Zhanjiang China; ^2^ Southern Marine Science and Engineering Guangdong Laboratory (Zhanjiang) Zhanjiang China; ^3^ Marine Biomedical Research Institution Guangdong Medical University Zhanjiang China

**Keywords:** agarophytes, coastal ecosystems, molecular markers, morphology, newly recorded species, species richness

## Abstract

This study was conducted to assess the diversity of Gracilariaceae species across various coastal ecosystems in Zhanjiang, Guangdong Province, China, and identify species suitable for large‐scale cultivation in the southern coastal waters of China. The diversity and seasonal and spatial distribution patterns of Gracilariaceae species in different ecosystems were systematically analyzed, and taxonomic studies were performed on species with disputed taxonomic identities using morphological and multi‐gene marker techniques to clarify their classification status. Species richness was higher, but individual species coverage was lower in open ecosystems (e.g., tidal pools) compared to enclosed ecosystems (e.g., mangroves, seagrass beds, saltwater ponds), and both factors showed significant seasonal variation. Conversely, enclosed ecosystems had lower species richness, higher species coverage, and minimal seasonal variation. The presence of *Gracilaria fisheri* in China was recorded for the first time, and the taxonomic status of *G. hainanensis* was systematically evaluated. Based on taxonomic evaluations and a review of the literature, *G. changii* and *G. firma* were proposed to be synonymous. In total, eight Gracilariaceae species were identified during the survey. Among them, *Gracilariopsis heteroclada*, *G. fisheri* , *G. edulis* , and *G. hainanensis* were identified as potential candidates for large‐scale cultivation in the southern coastal waters of China. This study advanced the understanding of the taxonomy and ecology of Gracilariaceae species in the Zhanjiang region and provided a scientific foundation for the conservation and industrial development of Gracilariaceae resources.

## Graphical abstract

This study assessed the diversity and distribution of Gracilariaceae species across various coastal ecosystems in Zhanjiang, Guangdong Province, China, identifying species suitable for large‐scale cultivation in southern China's coastal waters. Eight species were identified, with Gracilariopsis heteroclada, *Gracilaria fisheri*, *G. edulis* , and *G. hainanensis* proposed as potential candidates for cultivation. The research also provided new insights into the taxonomy, ecology, and conservation of Gracilariaceae species in the region.

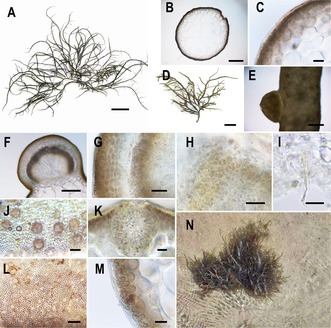

## 1 Introduction

The family Gracilariaceae belongs to the phylum Rhodophyta (red algae), class Florideophyceae, and order Gracilariales. *Gracilaria* Greville 1830 (hereafter *G*.) and *Gracilariopsis* E.Y. Dawson 1949 (hereafter *Gp*.) are the two most widely distributed genera in this family (which currently include 206 and 23 taxonomically accepted species, respectively), and are distributed from the northern to the southern coastal waters in China (Guiry 2024). They play critical roles as primary producers in marine ecosystems and also serve as essential sources of agar and are frequently used as feed for abalone, which highlights their significant economic value (Armisen 1995; Murano 1995; Francavilla et al. 2013). Since the late twentieth century, Chinese researchers have conducted extensive studies of *Gracilariopsis lemaneiformis* (Bory) E. Y. Dawson, Acleto & Foldvik 1964, cultivating multiple strains and achieving large‐scale cultivation along the northern and eastern coasts of China. This success has established China as one of the world's leading agar producers (Yang et al. 2015; Wang et al. 2020). However, *Gp. lemaneiformis*, which is relatively more suitable for growth in colder environments, is currently cultivated in China only as far south as Nan'ao Island in Shantou, Guangdong. It is unsuitable for large‐scale cultivation in warmer southern coastal waters, such as those in Zhanjiang and Hainan (Zhou et al. 2013). This limitation has hindered the expansion of Gracilariaceae farming in southern regions, despite the growing global demand for Gracilariaceae‐derived products. Therefore, identifying Gracilariaceae species suitable for large‐scale cultivation in southern China is crucial for expanding cultivation areas and fostering the development of the national Gracilariaceae industry.

Zhanjiang is located in southwestern Guangdong Province at the southernmost tip of mainland China. It is surrounded by the sea on three sides and has a coastline exceeding 2000 km (Zhang et al. 2010). The diverse coastal ecosystems in this region provide ideal habitats for various Gracilariaceae species. Research on Gracilariaceae resources in Zhanjiang's coastal areas began in the late twentieth century, and *Gracilaria firma* C. F. Chang and B. ‐M. Xia 1976 and *G. mixta* I. A. Abbott, J. Zhang and B. M. Xia 1991 were both discovered and named in this region (Chang 1976; Abbott et al. 1991). Zhang et al. (2014) surveyed macroalgal diversity as well as seasonal and spatial variations in several mangrove areas, identified two Gracilariaceae species, and recorded their ecological characteristics. Li et al. (2023) used morphological and molecular studies to supplement the taxonomic information available for four Gracilariaceae species located in Zhanjiang. These studies significantly advanced the conservation and utilization of Gracilariaceae resources in Zhanjiang, but notable limitations remain. For example, Zhang et al. (2014) focused exclusively on mangrove areas, leaving other coastal ecosystems underexplored. Additionally, our field surveys indicate that the diversity of Gracilariaceae species in Zhanjiang exceeds the four species documented by Li et al. (2023), and some species remain unidentified (unpublished data), highlighting the need for additional taxonomic studies. Furthermore, the region hosts several morphologically similar species, such as *G. hainanensis* C. F. Chang and B. M. Xia 1976, *G. firma*, *G. changii* (B. M. Xia and I. A. Abbott) I. A. Abbott, J. Zhang and B. M. Xia 1991, and *G. blodgettii* Harvey 1853, which are frequently misidentified, leading to classification errors. These misidentifications, resulting from morphological methods, cause molecular sequences to be uploaded to GenBank under incorrect names. Such erroneous sequences are then used by subsequent researchers, further perpetuating taxonomic confusion (Ng et al. 2017; Li et al. 2023; Wang et al. 2023). These research gaps and taxonomic inaccuracies hinder the healthy development of the Gracilariaceae industry in the region.

To address these gaps in our knowledge of Gracilariaceae species in Zhanjiang, Guangdong Province, we systematically surveyed their diversity across various coastal ecosystems in the region. Seasonal and spatial distribution patterns were analyzed, and taxonomic ambiguities were resolved using a combined morphological and molecular approach. Our findings not only enhanced our understanding of Gracilariaceae diversity but also identified potential species for large‐scale cultivation in southern China, which will support the sustainable utilization of these resources.

## 2 Materials and Methods

### 2.1 Study Sites

**FIGURE 1:**
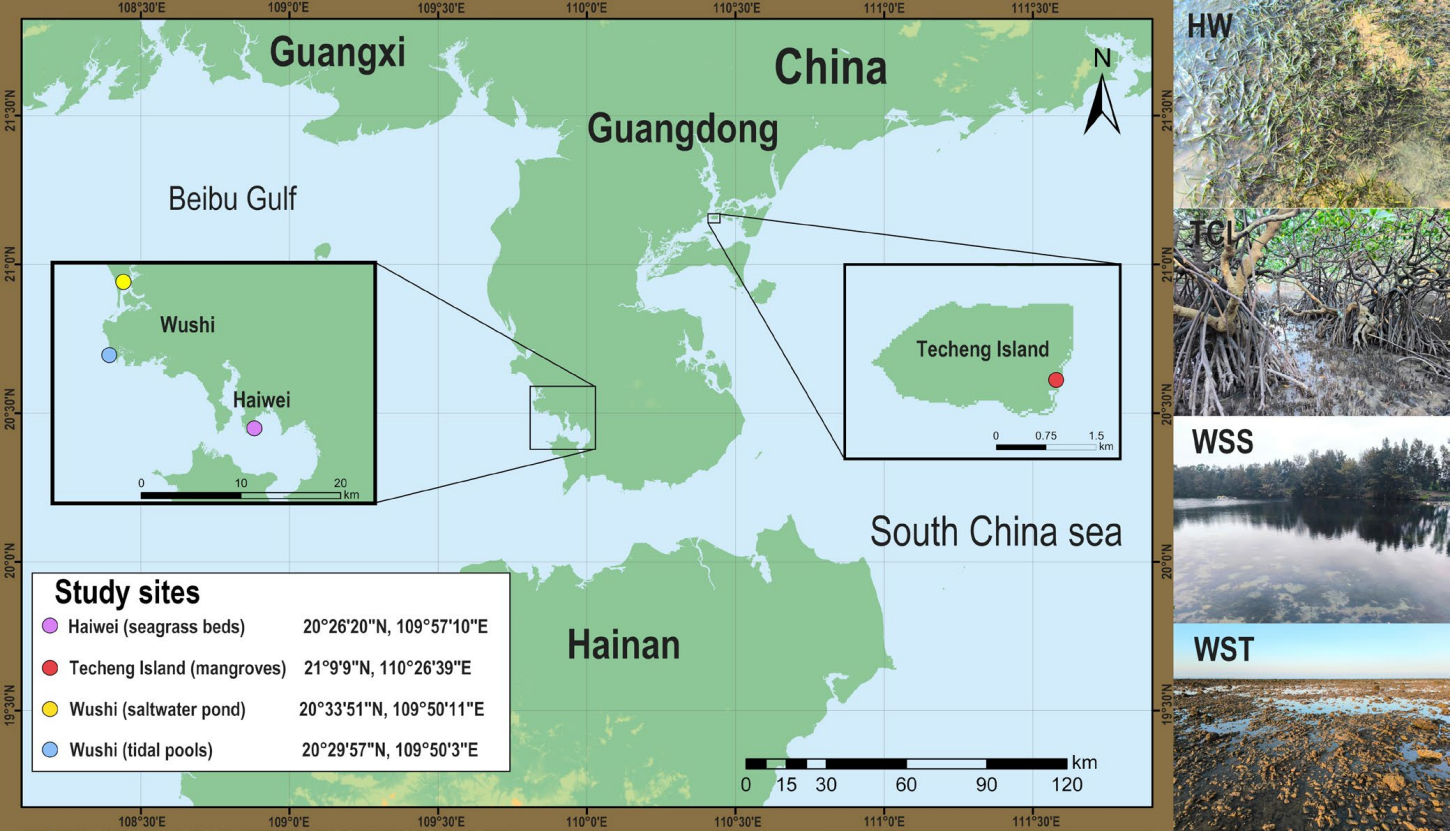
Study sites and habitats. The map displays the four representative coastal ecosystems (represented by colored dots) selected for in‐depth investigation in Zhanjiang, Guangdong Province, China. The habitat images on the right, from top to bottom, show the following locations: Seagrass beds in Haiwei (HW, 20°26′20″ N, 109°57′10″ E); mangroves of Techeng Island (TCI, 21°9′9″ N, 110°26′39″ E); saltwater ponds of Wushi (WSS, 20°33′51″ N, 109°50′11″ E); and tidal pools of Wushi (WST, 20°29′57″ N, 109°50′3″ E).

We selected four representative coastal ecosystems in Zhanjiang, Guangdong Province, China, for in‐depth investigation based on their ecological diversity and significance: Techeng Island mangroves (TCI), Haiwei seagrass beds (HW), Wushi tidal pools (WST), and saltwater ponds (WSS) (Figure 1). To ensure comprehensive data collection, study sites were subdivided by tidal influence and habitat characteristics. In WST and HW, surveys were conducted in high, middle, and low tide zones, labeled as tidal pool (TP)‐HT, TP‐MT, TP‐LT, seagrass bed (SB)‐HT, SB‐MT, and SB‐LT, respectively. Two zones were studied in the TCI area [mangroves (MG) and the edge of mangroves (EMG)] to capture both the core and transitional zones of the ecosystem. In total, nine sampling locations were sampled in four seasons.

### 2.2 Survey Methods and Sample Collection

To assess the seasonal coverage of Gracilariaceae species, surveys were conducted in autumn (September–October 2023), winter (December 2023–January 2024), spring (March–April 2024), and summer (July–August 2024) in TCI, HW, WST, and WSS. The survey methods were designed and implemented with reference to the national standard *Specifications for Oceanographic Survey—Part 6: Marine Biological Survey* (GB/T 12763.6‐2007) and the Shenzhen local standard *Technical Specification for Seaweed Bed Survey* (DB4403/T 394—2023).

We employed a belt transect method and established 50 × 20 m transects at each site; transects were spaced at least 50 m apart to ensure sampling independence. Within each transect, 25 × 25 cm quadrats were randomly placed by tossing to ensure unbiased data collection. Each quadrat was tossed a predetermined number of times along the transect line, with standardized angles and distances to minimize researcher bias (English et al. 1997). The coverage of each Gracilariaceae species within each quadrat was visually estimated and measured with a ruler for verification (Dethier et al. 1993). Photographs of each quadrat were taken using a Canon EOS M6 camera (Canon, Tokyo, Japan) for further post‐field analysis and consistency in species identification.

Gracilariaceae specimens were collected within quadrats using knives, scissors, and forceps. Samples were carefully removed from substrates (e.g., rocks, gravel, shells) with minimal thallus damage to facilitate accurate morphological identification (Zhang et al. 2014). The samples were transported to the laboratory under refrigeration at 4°C. Upon arrival, they were rinsed with sterilized seawater to remove surface impurities and immediately processed for morphological observation. Each study site was surveyed in triplicate, with three replicate quadrats at each point to ensure data reliability and reproducibility (Underwood 1997).

### 2.3 Identification of Gracilariaceae Species

#### 2.3.1 Morphological Identification

All collected samples were subjected to morphological identification. For species with clear and distinguishable morphological features, identification was based directly on characteristics such as long secondary branches, short needle‐like tertiary branches, clavate internodes, and very slender, profusely branched thalli (Xia and Zhang 1999; Li et al. 2023). In fact, our research team has previously conducted multiple macroalgae resource surveys in the Zhanjiang region. Most of the species collected for this study were previously reported by two of the authors, Li et al. (2023) and Zeng et al. (2025), and thus are not presented again in detail in this paper.

For species not covered in our previous studies or those whose taxonomic identity could not be clearly determined through morphology, we followed the descriptive methods of Hoek et al. (1995) and Xia and Zhang (1999). The morphological characteristics of these specimens were recorded in detail using a Canon EOS M6 camera, including features such as thallus color, texture, shape, branching pattern, and branch base constrictions. Additionally, hand‐cut transverse sections were prepared and observed under a light microscope (Olympus CX33, Tokyo, Japan) to examine the internal structure of the samples, including the size and shape of cortical and medullary cells, as well as reproductive structures (cystocarps, spermatangia, and tetrasporangia). To ensure long‐term preservation, herbarium specimens were prepared from selected samples for archival purposes and deposited in the Aquatic Organisms Museum of Guangdong Ocean University.

#### 2.3.2 *Cox*1 and *rbc*L Gene Sequence Analysis

Total DNA was extracted from morphologically indistinguishable Gracilariaceae samples using the Rapid Plant Genomic DNA Isolation Kit (Sangon Biotech, Shanghai, China) and stored at −20°C. PCR amplification of *rbc*L sequences was performed using the primers F57/R1381 (Freshwater and Rueness 1994), F7/R753, and F645/RrbcSstart (Lin et al. 2001; Gavio and Fredericq 2002), and *Cox*1 sequences were amplified with GWSFn/CoxIR1 (Saunders 2008) primers. The PCR program included an initial denaturation at 94°C for 3 min followed by 35 cycles of denaturation at 94°C for 30 s, annealing at 49.5°C for 40 s, and extension at 72°C for 50 s (*Cox*1) or 90 s (*rbc*L), with a final extension at 72°C for 5 min. PCR products were visualized on 1% agarose gels, cloned, and sequenced by Sangon Biotech (Guangzhou, China). The *Cox*1 and *rbc*L genes were selected because they are the most commonly used markers for identifying species within the Gracilariaceae family.

To provide a more comprehensive comparison of phylogenetic relationships, three methods were used to construct *rbc*L and *Cox*1 phylogenetic trees in this study: Neighbor‐Joining (NJ), Maximum Likelihood (ML), and Bayesian Inference (BI). For all analyses, *Rhodymenia pseudopalmata* (J. V. Lamouroux) P. C. Silva (1952) was used as the outgroup. Sequences, including those amplified in this study (Table 1) and retrieved from GenBank (Tables S1 and S2), were aligned and refined using BioEdit v7.2.6.1 and analyzed in MEGA v11.0 (Tamura et al. 2004). Optimal evolutionary models were determined with Modeltest v3.7 (Posada and Buckley 2004). The NJ tree was constructed using the Kimura two‐parameter model, the ML tree was built using the General Time Reverse (GTR) + gamma distribution (G) + invariable sites (I) model, and the BI tree was generated using the GTR model. Subsequent analyses were performed using MEGA v11.0 for the NJ and ML trees, with 1000 bootstrap replicates for both analyses. The BI tree was generated in MrBayes v3.1.2 (Ronquist and Huelsenbeck 2003), running for 1,000,000 generations with a sampling interval of 1000 generations, discarding the first 25% of trees as burn‐in. Genetic distances were calculated using the Kimura two‐parameter model in MEGA v11.0 to assess interspecific divergence.

**TABLE 1:** Sample information for *rbc*L and *Cox*1 sequences used in this study.

Strain code	Collection locality	Collection date	Accession no. of *rbc*L	Accession no. of *Cox*1
WST001	Wushi, Guangdong, China	8‐Mar‐2024	/	PQ818019
WST002	Wushi, Guangdong, China	8‐Mar‐2024	PQ818037	PQ818020
WST003	Wushi, Guangdong, China	8‐Mar‐2024	/	PQ818021
WST004	Wushi, Guangdong, China	8‐Mar‐2024	PQ818038	PQ818022
WST005	Wushi, Guangdong, China	8‐Mar‐2024	PQ818039	PQ818023
WST006	Wushi, Guangdong, China	25‐Jul‐2024	/	PQ818024
WST007	Wushi, Guangdong, China	25‐Jul‐2024	/	PQ818025
WST008	Wushi, Guangdong, China	25‐Jul‐2024	/	PQ818026
WST009	Wushi, Guangdong, China	25‐Jul‐2024	/	PQ818027
WST010	Wushi, Guangdong, China	25‐Jul‐2024	/	PQ818028
WST011	Wushi, Guangdong, China	25‐Jul‐2024	/	PQ818029
WST012	Wushi, Guangdong, China	25‐Jul‐2024	/	PQ818030
WST013	Wushi, Guangdong, China	25‐Jul‐2024	/	PQ818031
WST014	Wushi, Guangdong, China	25‐Jul‐2024	/	PQ818032
WST015	Wushi, Guangdong, China	25‐Jul‐2024	/	PQ818033
TCI001	Techeng Island, Guangdong, China	20‐Aug‐2024	PQ818040	PQ818034
TCI002	Techeng Island, Guangdong, China	20‐Aug‐2024	PQ818041	PQ818035
TCI003	Techeng Island, Guangdong, China	20‐Aug‐2024	/	PQ818036
TCI005	Techeng Island, Guangdong, China	20‐Aug‐2024	PQ818042	/
TCI006	Techeng Island, Guangdong, China	20‐Aug‐2024	PQ818043	/

*Note:* “/” stands for data not available.

### 2.4 Diversity Analysis

#### 2.4.1 Species Diversity Analysis

To quantify the species diversity of Gracilariaceae across the nine sampling locations and four seasons, we calculated the Shannon diversity index (*H′*) and the Simpson diversity index (*D*) using species coverage data from each site (Simpson 1949; Magurran 2003) as follows:

$$H^{\prime }=-\sum_{i=1}^{S}p_{i}\ln\left(p_{i}\right)$$

$$D=1-\sum_{i=1}^{S}p_{i}^{2}$$

where *S* is the number of species, and *p*_*i*_ represents the proportion of the coverage of species *i* relative to the total coverage across all replicates within each site and season.

Both indices were calculated using R v4.4.1 (Zuur et al. 2007) with the vegan v2.6.8 package (Oksanen 2010). Results were visualized with ggplot2 v3.5.1, and boxplots illustrated diversity patterns across seasons and locations (Wickham and Wickham 2016). The normality of the data was tested using the Shapiro–Wilk test, which indicated non‐normality for all diversity indices (*W* = 0.82–0.91, *p* < 0.05). Therefore, a separate Kruskal‐Wallis test was employed to assess the significance of differences in diversity indices among seasons and locations (McCune and Grace 2002). The Kruskal‐Wallis test statistic (*χ* ^2^) and its associated degrees of freedom (df) were reported to quantify the magnitude of inter‐group differences, with *p*‐values indicating the probability of observing such differences under the null hypothesis. Post hoc Dunn's test using dunn.test v1.3.6 was applied for post hoc pairwise comparisons within each factor group (ecosystems or seasons). All statistical analyses were conducted with a significance level of *α* = 0.05.

#### 2.4.2 Species Coverage Analysis

To assess the spatial and temporal variation in species coverage of Gracilariaceae across the nine sampling locations and four seasons, we calculated the average coverage of each species across replicates within each site and season (Kent 2011). These data were visualized as a heatmap using the pheatmap v1.0.12 package in R v4.4.1, illustrating species coverage patterns across different sites and seasons (Kolde and Kolde 2015). Prior to statistical analysis, the normality of the species coverage data was tested using the Shapiro–Wilk test, which showed non‐normality (*W* = 0.79–0.88, *p* < 0.05). A separate Kruskal‐Wallis test was used to assess significant differences in species coverage between the sites and seasons (McCune and Grace 2002). For post hoc analysis, Dunn's test was employed to perform pairwise comparisons between groups to determine specific significant differences. All analyses were conducted using R, with the dunn.test package for post hoc comparisons. All statistical analyses were conducted with a significance level of *α* = 0.05.

## 3 Results

### 3.1 Identification of Gracilariaceae Species and Distribution

#### 3.1.1 Composition of Gracilariaceae Species Across Distinct Ecosystems in Zhanjiang

Based on the Gracilariaceae samples collected from different ecosystems in Zhanjiang, a total of eight species belonging to two genera were identified (Table 2). Six of these species have distinct morphological characteristics, namely *Gp. heteroclada* J. ‐F. Zhang and B. ‐M. Xia 1991, *G. salicornia* (C. Agardh) E. Y. Dawson 1954, *G. tenuistipitata* C. F. Chang and B. ‐M. Xia 1976, *G. vermiculophylla* (Ohmi) Papenfuss 1967, *G. edulis* (S. G. Gmelin) P.C. Silva (1952), and *G. firma*, which could be directly identified by referring to Xia and Zhang (1999) and our team's published work (Li et al. 2023). The remaining two species ( *G. fisheri* (B. M. Xia and I. A. Abbott) I. A. Abbott, J. Zhang and B. M. Xia 1991 and *G. hainanensis* ) could not be identified based on initial morphological assessment; therefore, we provided detailed morphological descriptions and employed multi‐gene marker techniques for their identification. Five, four, two, and two species were found in WST, HW, WSS, and TCI, respectively. *G. salicornia* and *Gp. heteroclada* exhibited broad distribution and occurred in multiple ecosystems. In contrast, *G. fisheri* , *G. vermiculophylla*, *G. edulis* , and *G. hainanensis* were restricted to specific ecosystems.

**TABLE 2:** Species of Gracilariaceae found in Zhanjiang in this study.

Genus	Species	Morphological features	HW	WSS	WST	TCI
*Gracilariopsis*	*Gp. heteroclada*	Long secondary branches, short needle‐like tertiary branches	x	x	x	
*Gracilaria*	*G. salicornia*	Clavate internodes	x	x	x	
	*G. tenuistipitata*	Very slender thallus, profusely branched	x			
	*G. vermiculophylla*	Slender thallus				x
	*G. edulis*	Corymbose/brush‐like appearance, with creeping stolons, reddish branch bases			x	
	*G. fisheri*	Corymbose/brush‐like appearance, branch bases slightly tapered			x	
	*G. firma*	Light green, small thallus, highly constricted branch bases	x		x	
	*G. hainanensis*	Dark red, large thallus, highly constricted branch bases				x

*Note:* An ‘x’ indicates the presence of a species in a given environment. Each environment is shown in a separate column.

#### 3.1.2 Morphological Description of the Newly Recorded Species in China: *G. fisheri*

**FIGURE 2:**
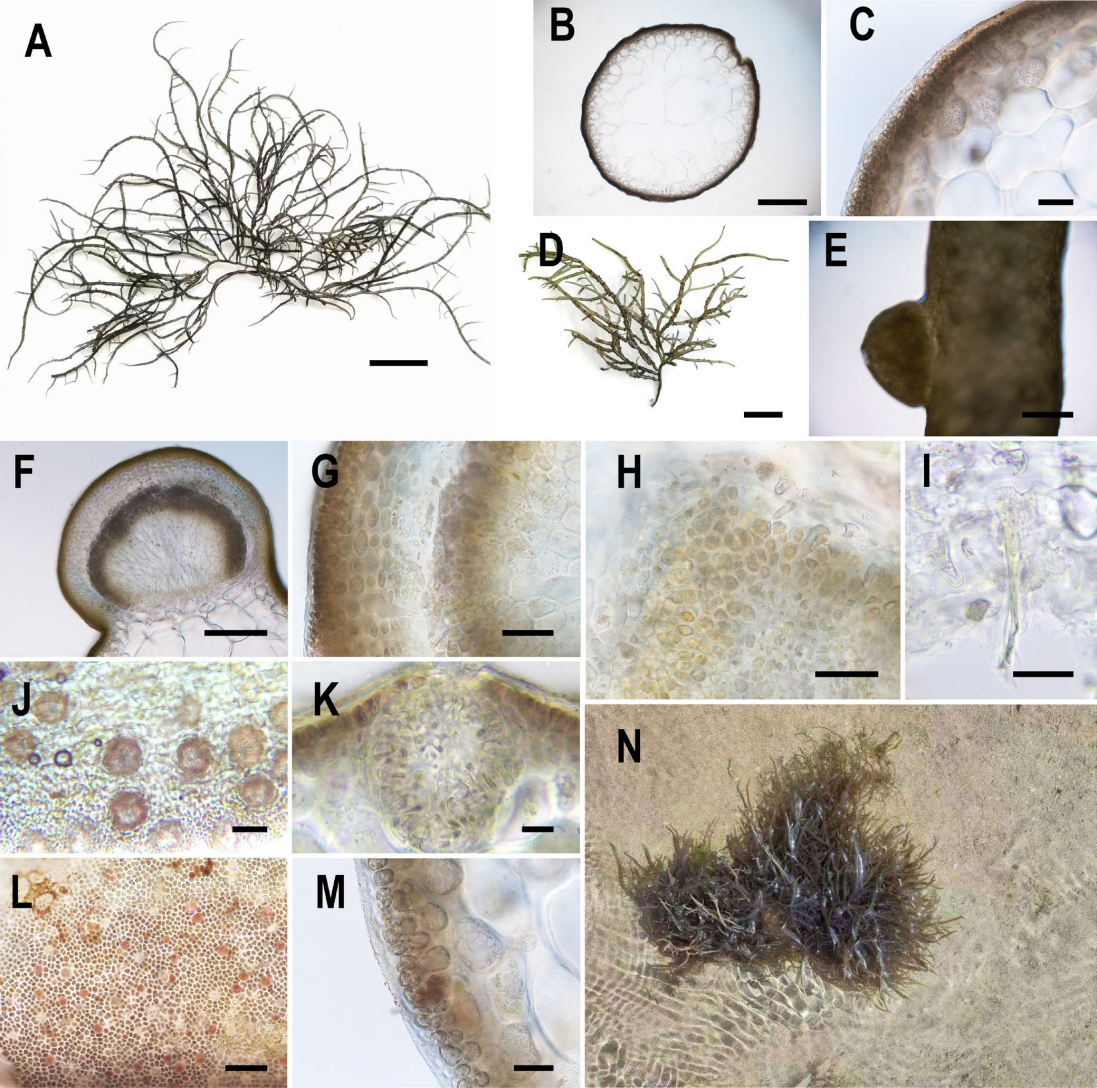
Morphology of *Gracilaria fisheri* with its habitat. (A) Thallus morphology; (B) Cross‐section of thallus. (C) Enlarged view of thallus cross‐section. (D) Cystocarps densely distributed on the thallus surface. (E) External view of a cystocarp. (F) Cross‐section of a cystocarp. (G) Cross‐section of the cystocarp wall. (H) Carpospores; (I) Absorbent filaments. (J) Surface view of spermatangia. (K) Cross‐section of spermatangia. (L) Surface view of tetrasporangia. (M) Cross‐section of tetrasporangia. (N) Habitat of *G. fisheri* , growing in tidal pools. Scale bars: A = 4 cm; B, E = 400 μm; C = 100 μm; D = 2 cm; F = 200 μm; G, J, L = 40 μm; H = 150 μm; I, K = 20 μm; M = 50 μm.

The thallus of *G. fisheri* is erect, cylindrical, and can be solitary or form clusters; it is anchored by a disc‐shaped holdfast at the base. Thallus height ranges from 10 to 35 cm and varies seasonally: 10–18 cm in late autumn, late spring, or early summer, and 15–35 cm in winter and spring. The main axis measures 0.5–2 mm in width and is visible only near the base (Figure 2A). Numerous lateral branches are present and mostly irregularly opposite, with some alternate branches, and branch spacing decreases towards the upper thallus. In smaller individuals collected during summer and autumn, the branches are short and concentrated at the apices. Thallus coloration is highly variable; it is typically light brown or yellow‐brown, but greenish individuals are occasionally collected in summer and autumn. Pressed specimens adhere well to paper.

Cross‐sectional analysis (Figure 2B,C) revealed that the medulla consisted of large, colorless, nearly spherical parenchymatous cells with diameters ranging from 160 to 550 μm, and cell walls are 3.5–6 μm thick. The cortex consisted of 3–5 cell layers, with individual cell diameters between 5 and 50 μm. The outer 1–2 layers contained smaller pigmented cells. A distinct boundary existed between the medulla and cortex, and the surface was covered by a gelatinous layer that was 4–7 μm thick.

Cystocarps densely covered the thallus surface (Figure 2D) and measured 700–1200 μm in height and 1300–1800 μm in width. They were prominently protruding and hemispherical or conical in shape, and they lacked a distinct beak or possessed an indistinct one. The base was either unconstricted or slightly constricted (Figure 2E,F). The cystocarp wall consisted of 7–10 cell layers, with a total thickness of 120–180 μm (Figure 2G). The carposporophyte contained spherical to ovoid carpospores, each measuring 20–30 μm in diameter (Figure 2H). Short, colorless, transparent absorbent filaments were present between the cystocarp wall and gonimoblast filaments, but they were difficult to detect (Figure 2I).

Spermatangia densely populated the cortical cells. They appeared colorless with strong reflectivity, and they were irregularly round in surface view (Figure 2J). They measured 40–63 × 43–50 μm in cross‐section. Spermatangial chambers were composed of multiple spherical or ovoid cavities, classified as “P” type, with openings at the apex of the spermatangia (Figure 2K). Tetrasporangia were densely distributed within the cortex. They were ovoid to elongated in surface view and displayed a reddish‐brown color when mature but were colorless when immature (Figure 2L). In cross‐section, tetrasporangia were ellipsoidal, measuring 25–30 μm in height and 9–15 μm in width, and they featured a distinct cruciate division (Figure 2M).

In this study, *G. fisheri* frequently formed large clusters during winter and spring, thriving in tidal pools with sandy and rocky substrates within the intertidal zone. After tide recession, the species remained submerged in water depths ranging from 5 to 40 cm (Figure 2N). It often co‐occurred with *G. edulis* .

Specimen information: WST001‐WST010, collected by Zhaojun Zeng on March 8 and July 25, 2024, from Wushi, Zhanjiang, Guangdong Province, China. The specimens are currently deposited in the Aquatic Organisms Museum of Guangdong Ocean University.

#### 3.1.3 Morphological Description of *G. hainanensis* Found in Mangroves

**FIGURE 3:**
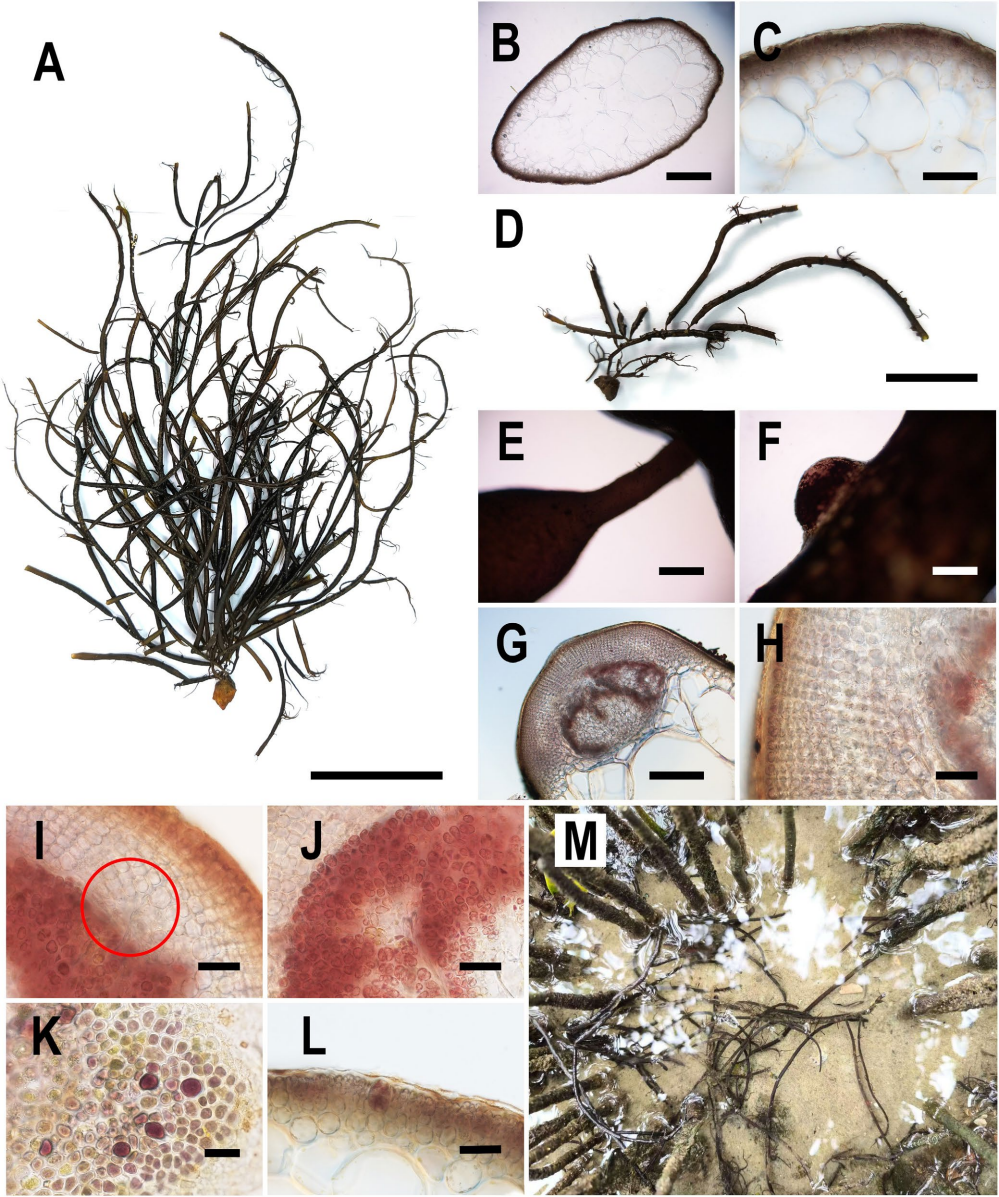
Morphology of *Gracilaria hainanensis* with its habitat. (A) Thallus morphology. (B) Cross‐section of thallus. (C) Enlarged view of thallus cross‐section. (D) Cystocarps scattered on the thallus surface. (E) Highly constricted branch bases. (F) External view of a cystocarp. (G) Cross‐section of a cystocarp. (H) Cross‐section of the cystocarp wall. (I) Absorbent filaments. (J) Carpospores. (K) Surface view of tetrasporangia. (L) Cross‐section of tetrasporangia. (M) Habitat of *G. hainanensis* , with branches entwining around mangrove roots. Scale bars: A = 4 cm; B, E, F = 400 μm; C, H, I, J, L = 40 μm; D = 2 cm; G = 200 μm; K = 20 μm.

The thallus of *G. hainanensis* was erect and cylindrical and predominantly solitary, with few instances of being clustered. It tapered slightly near the base and was anchored by a small disc‐shaped holdfast. Individuals were relatively large, generally ranging from 20 to 50 cm in height, with some specimens exceeding 60 cm. The main axis measured 1–4 mm in width. Branching was predominantly irregularly opposite or alternate, with a very small number of dichotomous branches (Figure 3A). Branches ranged from 20 to 50 cm in length, with branch tips becoming gradually slender. However, most branches broke, forming cross‐sections that produced multiple small branches. Except for the few dichotomous branches, the base of other branches was highly constricted and formed a small stipe measuring 0.5–1.3 mm in length (Figure 3E). Thallus coloration was dark red, occasionally with branches exhibiting slight green or red hues. Fresh specimens were fleshy and brittle, whereas dried specimens became soft. Pressed specimens adhered completely to paper.

Cross‐sectional analysis of the thallus (Figure 3B,C) revealed that the medulla consisted of multiple large and irregular thin‐walled cells, with cell diameters ranging from 350 to 700 μm and wall thicknesses of 3.5–5.5 μm. The medulla at the lower part of the thallus often ruptured and became hollow. The cortex consisted of 2–4 cell layers composed of irregularly oval‐shaped cells with diameters between 5 and 25 μm. The outermost 1–3 layers of cells contained chloroplasts. A distinct boundary existed between the cortex and medulla. The surface was covered by a gelatinous layer 4.5–6.5 μm thick. The thallus surface was often adorned with various impurities and microalgae.

Cystocarps were scattered across the thallus surface (Figure 3D), measuring 300–450 μm in height and exhibiting hemispherical or conical shapes without a beak. The base was unconstricted (Figure 3F,G). The cystocarp wall consisted of 9–11 cell layers with a thickness of 80–140 μm (Figure 3H). Distinct absorbent filaments were visible between the gonimoblast filaments and the cystocarp wall (Figure 3I). Carpospores were spherical or oval and measured 15–30 μm in diameter (Figure 3J). Tetrasporangia exhibited cruciate division and are predominantly oval‐shaped or irregularly circular in surface view, with diameters ranging from 15 to 30 μm (Figure 3K). In cross‐section, tetrasporangia were ovate and measured 15–35 μm in height and 10–25 μm in width. They were a light reddish‐brown color. Epidermal cells surrounding the sporangia underwent metamorphosis and became elongated oval in shape (Figure 3L).

In this study, *G. hainanensis* was observed to grow throughout all seasons in mangroves with freshwater inflow. After tide recession, the species remained submerged in water depths ranging from 10 to 30 cm (Figure 3M). It commonly attached to sandy or gravel substrates and various shells, with branches often entwining around mangrove roots.

Specimen information: TCI001‐TCI005, collected by Zhaojun Zeng on Aug 20, 2024, from Techeng Island, Zhanjiang, Guangdong Province, China. The specimens are currently deposited in the Aquatic Organisms Museum of Guangdong Ocean University.

#### 3.1.4 *Cox*1 and *rbc*L Gene Sequence Analysis

**FIGURE 4:**
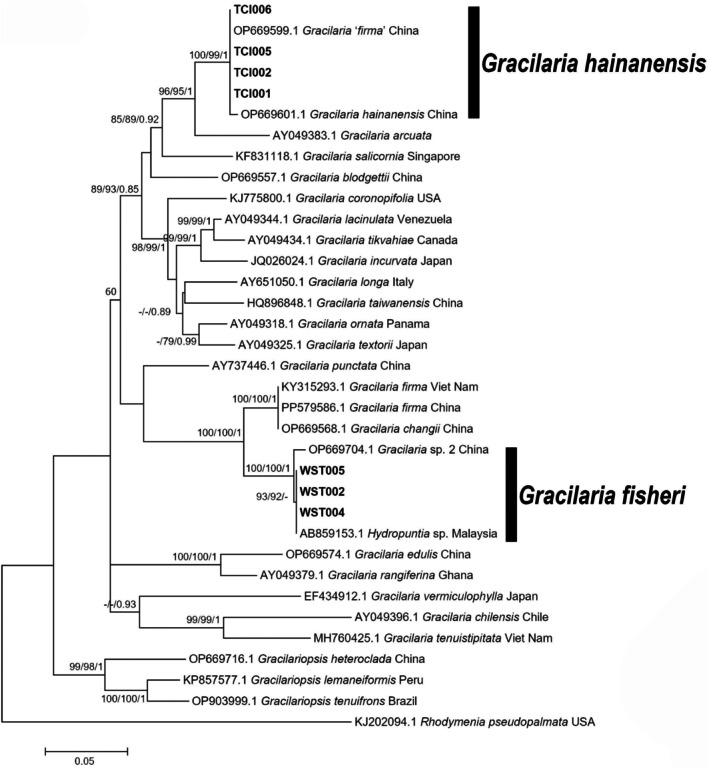
Maximum likelihood phylogenetic tree of Gracilariaceae species based on *rbc*L gene sequences. The scale bar indicates evolutionary distance. Bold branches represent sequences newly amplified in this study. Node values represent bootstrap support, shown as NJ/ML/BI from left to right. Only support values with Bayesian posterior probabilities ≥ 0.7 or bootstrap values (ML and NJ) ≥ 70 are displayed.

**FIGURE 5:**
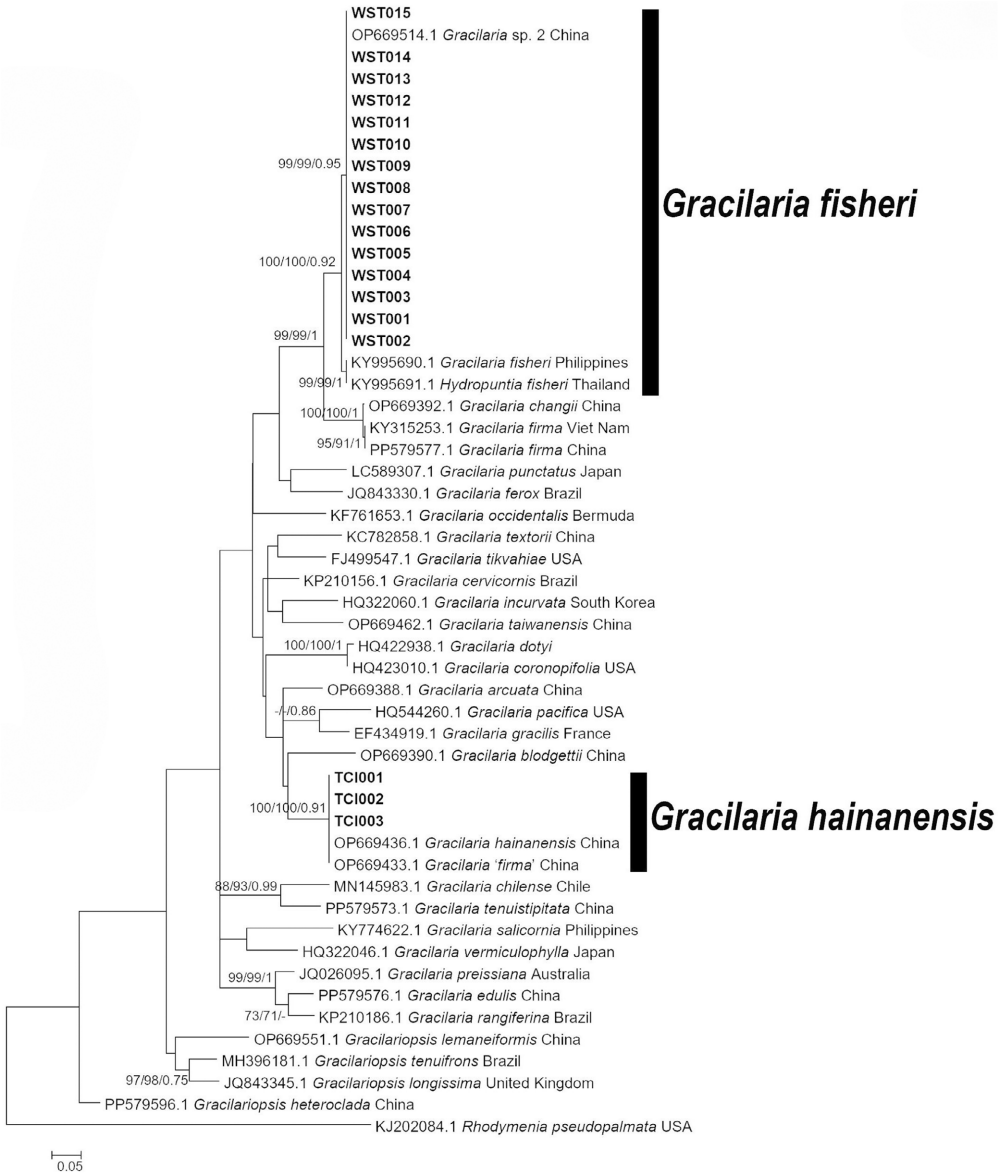
Maximum likelihood phylogenetic tree of Gracilariaceae species based on *Cox*1 gene sequences. The scale bar indicates evolutionary distance. Bold branches represent sequences newly amplified in this study. Node values represent bootstrap support, shown as NJ/ML/BI from left to right. Only support values with Bayesian posterior probabilities ≥ 0.7 or bootstrap values (ML and NJ) ≥ 70 are displayed.

A total of 35 *rbc*L sequences (seven newly generated in this study) and 52 *Cox*1 sequences (18 newly generated) were used to construct phylogenetic trees. The *rbc*L sequences were 1133 bp in length. The *Cox*1 sequences were 446 bp in length. Phylogenetic trees were constructed using NJ, ML, and BI methods. The three methods yielded consistent topologies, and the ML tree was selected for presentation (Figures 4 and 5), with node support values indicated as NJ/ML/BI.

In the *rbc*L phylogenetic tree, the seven sequences from this study formed two clades. The first clade included TCI001‐002 and TCI005‐006 and clustered with *G. hainanensis* (OP669601.1) and *G. ‘firma’* (OP669599.1) with strong support (100/99/1). These sequences showed no base differences, and the genetic distance was 0 from *G. ‘firma’* and 0.44% from *G. hainanensis* . The second clade included WST002, WST004, and WST005 and clustered with unidentified *Gracilaria* species from China (OP669704.1) and Malaysia (AB859153.1) with strong support (100/100/1). These sequences showed no base differences, and the genetic distance was 0 from *Hydropuntia* sp. and 0.8% from *Gracilaria* sp.2. Notably, the second clade appeared as a sister group to *G. firma* and *G. changii*, with strong support (100/100/1).

In the *Cox*1 phylogenetic tree, 18 sequences from this study also formed two clades. The first clade included TCI001‐003 and clustered with *G. hainanensis* (OP669436.1) and *G. ‘firma’* (OP669433.1) with strong support (100/100/0.91). These sequences showed no base differences and formed a single group with a genetic distance of 0 from both *G. hainanensis* and *G. ‘firma’*. The second clade included WST001‐015 and clustered with *Gracilaria* sp.2 (OP669514.1) from China. This clade was sister to another clade containing *G. fisheri* from the Philippines (KY995690.1) and Thailand (KY995691.1), with strong support (100/100/0.92). Notably, the second clade also appeared as a sister group to *G. firma* and *G. changii* with strong support (99/99/1), revealing the phylogenetic relationship between these two clades.

### 3.2 Diversity Analysis

#### 3.2.1 Species Diversity Analysis

**FIGURE 6:**
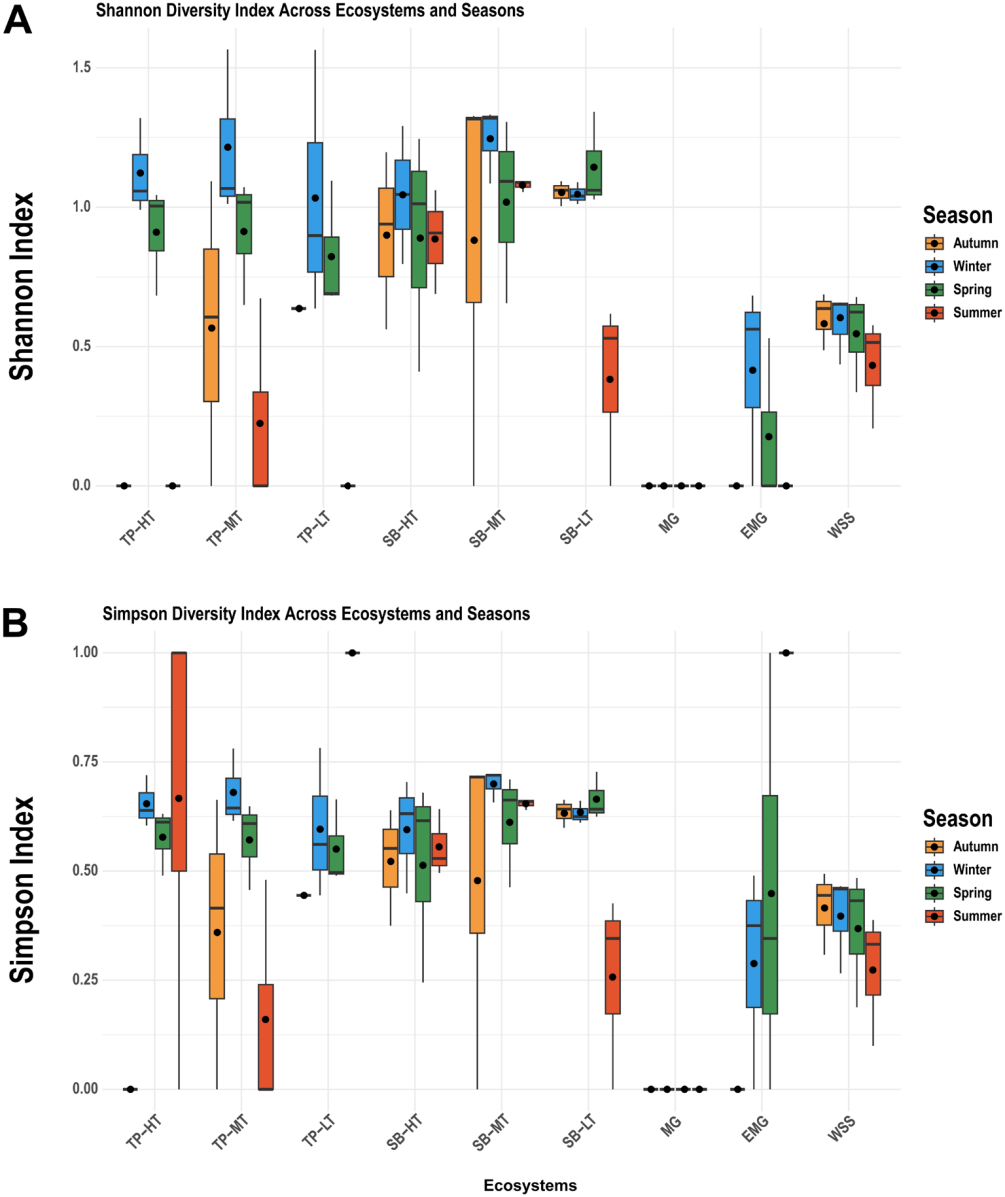
Boxplots of species diversity indices across different ecosystems and seasons. (A) Shannon diversity indices. (B) Simpson diversity indices. Boxes represent the interquartile range of the data, with the central line indicating the median and the dots indicating the mean.

Boxplots of the Shannon diversity index across various ecosystems and seasons reveal distinct diversity patterns (Figure 6A). The HW seagrass bed ecosystems (SB‐HT, SB‐MT, SB‐LT) consistently exhibited significantly higher Shannon diversity indices across all seasons (mean *H′* = 0.4–1.25) compared to those of the other ecosystems (Dunn's test, *p* < 0.05). In contrast, the TCI mangrove ecosystems (MG, EMG) displayed the lowest Shannon diversity indices (mean *H*′ = 0–0.2). Most ecosystems (excluding SB‐HT, SB‐MT, MG, and WSS) showed seasonal variations in Shannon diversity, with the lowest levels in summer (mean *H*′ = 0–0.88). The Kruskal‐Wallis test confirmed significant differences in Shannon diversity among ecosystems (*χ* ^2^ = 24.7, df = 8, *p* < 0.001) and seasons (*χ* ^2^ = 12.3, df = 3, *p* = 0.006), and post hoc Dunn's tests identified specific pairwise differences between ecosystems (e.g., SB‐HT vs. MG: *p* < 0.001) and seasons (summer vs. winter: *p* = 0.012).

The Simpson diversity index, reflecting species dominance, varies significantly among the studied groups (Figure 6B). The TCI mangrove ecosystems (MG, EMG) had the lowest Simpson diversity indices (mean *D* = 0–0.44), followed by the saltwater pond ecosystem (WSS, mean *D* = 0.28–0.42). The Kruskal‐Wallis test revealed significant differences among ecosystems (*χ* ^2^ = 18.9, df = 8, *p* < 0.001), but no significant seasonal variation was detected (*χ* ^2^ = 6.1, df = 3, *p* = 0.077).

#### 3.2.2 Species Coverage Analysis

**FIGURE 7:**
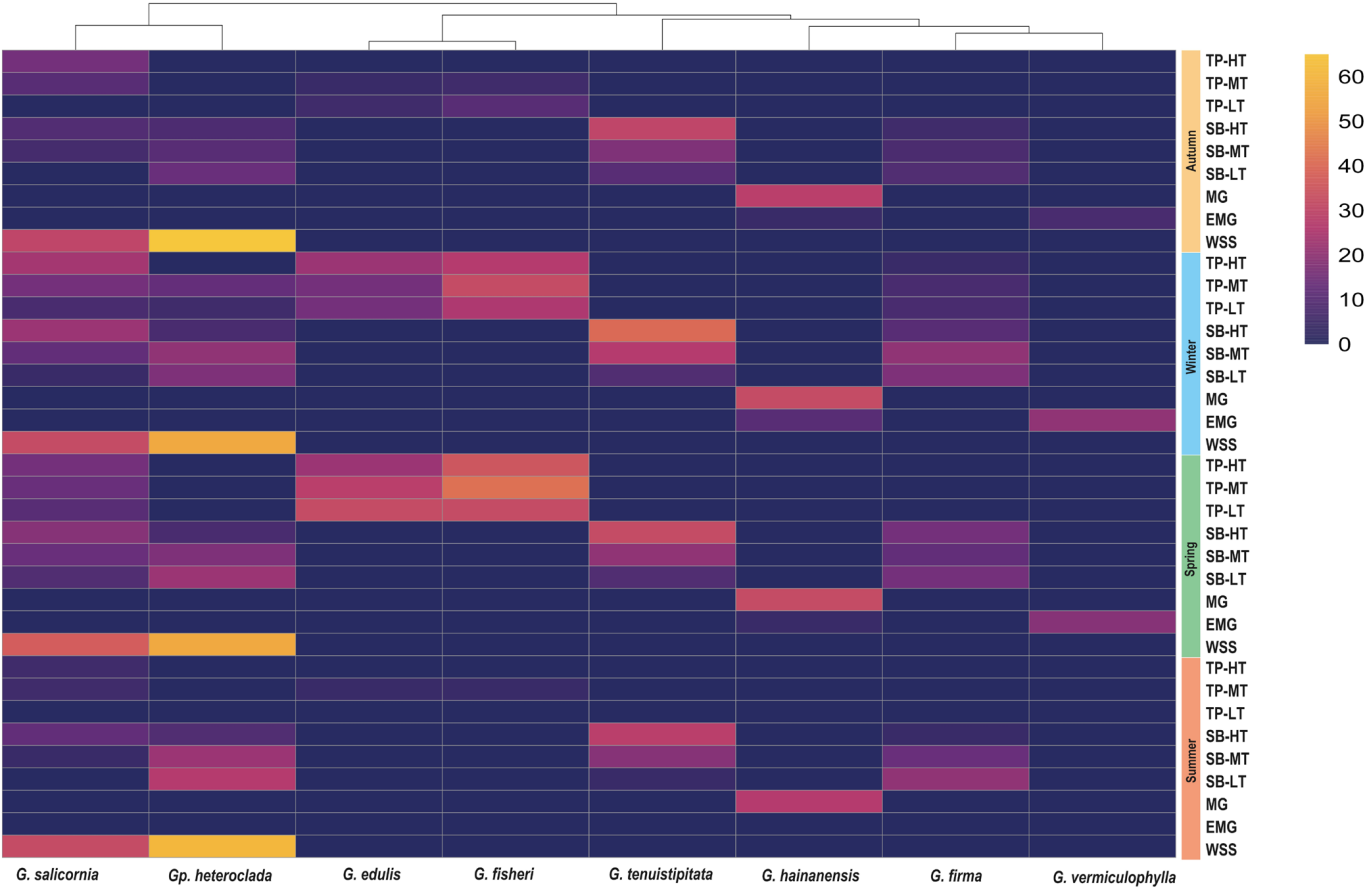
Species coverage heatmap for Gracilariaceae species across ecosystems and seasons. Species coverage data were averaged for each sampling location and season. The color gradient ranges from purple to yellow, representing increasing species coverage, with purple indicating lower coverage and yellow indicating higher coverage values.

Species coverage for the family Gracilariaceae exhibits notable spatial and temporal variations (Figure 7). *G. salicornia* was distributed in tidal pools, seagrass beds, and saltwater ponds across all seasons, with significantly higher coverage in saltwater ponds (mean = 30%–45%) compared to other ecosystems (Dunn's test, *p* < 0.05). *Gp. heteroclada* also exhibited widespread distribution in tidal pools, seagrass beds, and saltwater ponds year‐round, with exceptionally high coverage in saltwater ponds (exceeding 60% in all four seasons and reaching up to 70% in winter), with field observations indicating vertical stacking of algal mats.

*G. edulis* and G. *fisheri* showed significant seasonal variation in tidal pools (peak coverage in winter/spring: 25%–40%; summer: < 5%; Dunn's test, *p* < 0.05). G. *hainanensis* was exclusively detected in mangroves (MG: mean coverage = 26.7%–31.7%), with no significant seasonal differences (*χ* ^2^ = 2.1, df = 3, *p* = 0.55). Rare species (*G. firma*, *G. vermiculophylla*) maintained low coverage (< 15%) across ecosystems.

## 4 Discussion

### 4.1 Newly Recorded Species in China

*G. fisheri* was originally described by Xia and Abbott, who collected it from a Malaysian market and named it *Polycavernosa fisheri* B. M. Xia and I. A. Abbott 1987 (Bangmei and Abbott 1987) in honor of the algae specialist Jack Fisher. The species was later reclassified under *Hydropuntia* Montagne 1842 (Wynne 1989) and subsequently into the genus *Gracilaria* (Abbott et al. 1991). With the advent of molecular techniques, multiple studies have confirmed that *Polycavernosa*, *Hydropuntia*, and *Gracilaria* should be unified under the genus *Gracilaria* (Lyra et al. 2021). Historically, the presence of *G. fisheri* was reported in only a few Southeast Asian countries. In their study on Gracilariaceae diversity in China, Wang et al. (2023) identified a specimen (SY56‐1) with a *Cox*1 gene sequence showing 1.2% divergence from Thai populations of *G. fisheri* . However, morphologically, this specimen resembled *Gracilaria coronopifolia* J. Agardh 1852 more closely. Due to limited morphological comparisons, Wang et al. temporarily classified it as *Gracilaria* sp.2. In this study, samples WST001‐015 clustered with *Gracilaria* sp.2 in both the *rbc*L and *Cox*1 phylogenetic trees, with strong bootstrap support (100/100/1 for *rbc*L; 99/99/0.95 for *Cox*1). The genetic distances between these samples and *Gracilaria* sp.2 ranged from 0% to 0.8%, confirming their conspecific status. Additionally, in the *Cox*1 phylogenetic tree, these samples showed a genetic distance of only 1.36% from *G. fisheri* collected in the Philippines and Thailand and therefore were identified as sister taxa to *G. firma* or *G. changii*. These findings aligned with the phylogenetic results reported by Wang et al. (2023) and Ng et al. (2017).

To further confirm the taxonomic identity of WST001‐015, a detailed morphological analysis was conducted. Winter and early spring samples displayed a cymose or broom‐like thallus, measuring 15–35 cm in height, and they were light to yellow‐brown in color. Soralia were rare and inconspicuous, and the spermatangia were of the “P” type. Both the external morphology and internal structures, as well as the reproductive cell types, were consistent with the original description. These samples closely resembled the type specimen illustrated in Ng et al. (2017, figure 2, p. 5), further supporting their identification as *G. fisheri* .

The first occurrence of *G. fisheri* in China may be attributed to two possible factors. First, global warming might have facilitated the northward range expansion of this species, which is primarily distributed in Southeast Asia (Xia and Zhang 1999; Yang et al. 2015; Phang et al. 2016), into Chinese waters. A typical example of this expansion is *Ulva fasciata* Delile 1813 (now considered as *U. lactuca* Linnaeus 1753), which is a common species in the South China Sea (Huang et al. 2016). In recent years, its presence has also become more frequent in the East China Sea due to global warming. Second, historical misidentification could explain the lack of prior records for *G. fisheri* in China. In our study, samples collected in late autumn, late spring, or early summer were shorter (10–18 cm) and also cymose or broom‐like, and they closely resembled the description of *G. coronopifolia* by Xia and Zhang (1999). In previous surveys of the WST area, which lacked molecular analysis, these samples were often misidentified as *G. coronopifolia* . Notably, no molecular data for *G. coronopifolia* from China exist in GenBank, leaving its presence in China open to further investigation.

In this study, *G. fisheri* was frequently found growing intertwined with *G. edulis* during the same season. Although both species exhibit a cymose or broom‐like morphology, they could be distinguished by several key characteristics. *G. edulis* had prominent creeping stems, red coloration at the base of the branches, and a much rougher texture compared to *G. fisheri* (P. C. Silva 1952). These differences enabled clear differentiation between the two species during field surveys. Moreover, although the phylogenetic trees based on *rbc*L and *Cox*1 genes both indicated a close phylogenetic relationship between the clade of *G. fisheri* and the clade of *G. firma/G. changii*, significant morphological differences existed between them. The basal branches of *G. fisheri* were either not constricted or only slightly constricted, whereas the basal branches of *G. firma* and *G. changii* were highly constricted, forming a very small stipe (Xia and Zhang 1999).

### 4.2 Taxonomic Relationships of *Gracilaria* Species With Highly Constricted Branch Bases

The constriction and degree of constriction at branch bases are important morphological features for the taxonomic classification of Gracilariaceae. Compared to examining complex reproductive structures, these traits are more practical for preliminary species identification during field surveys. However, some scholars have overly relied on these features during taxonomic identification, neglecting other morphological characteristics. This has led to frequent misidentifications of *Gracilaria* species with severely constricted branch bases and controversies regarding their taxonomic relationships. Currently, five *Gracilaria* species with severely constricted branch bases have been reported in China (Xia and Zhang 1999): *G. changii* (B. M. Xia and I. A. Abbott) I. A. Abbott, J. Zhang and B. M. Xia 1991; *G. firma* C. F. Chang and B. ‐M. Xia 1976; *G. hainanensis* C. F. Chang and B. M. Xia 1976; *G. blodgettii* Harvey 1853; and *G. mixta* I. A. Abbott, J. Zhang & B. M. Xia 1991. Among them, the taxonomic relationships of the first four remain contentious, whereas *G. mixta* has rarely been studied.

Historically, scholars often identified *Gracilaria* species with highly constricted branch bases as *G. blodgettii* , resulting in species such as *G. firma* or *G. changii* being misidentified as *G. blodgettii* . This misidentification not only led to morphological errors but also caused incorrect genetic sequences to be uploaded to the GenBank database. Ng et al. (2017), Li et al. (2023), and Wang et al. (2023) have noted that some *G. blodgettii Cox*1 sequences from China and the Philippines in the GenBank database lack proper documentation and are evidently erroneous. These flawed sequences not only caused confusion among the sequences of multiple species in the database but also exacerbated species identification errors. For instance, Hassan et al. (2019), when constructing a *Cox*1 phylogenetic tree, utilized these erroneous sequences, leading to their *G. firma* or *G. changii* samples being misidentified as *G. blodgettii* , thereby further compounding the confusion in the GenBank database. In fact, the “T”‐shaped spermatangia of *G. blodgettii* are morphologically distinct from the “V”‐shaped and “P”‐shaped spermatangia of *G. firma* and *G. changii*, respectively (Harvey 1853; Xia and Zhang 1999). If taxonomists had conducted a more comprehensive morphological comparison, these identification errors could have been avoided.

To clarify the taxonomic relationship between *G. firma* and *G. changii*, Ng et al. (2017) analyzed samples of *G. firma* and *G. changii* collected from multiple Southeast Asian countries and Taiwan using morphological and multi‐gene markers. They concluded that *G. firma* and *G. changii* are the same species. Phylogenetic trees based on *rbc*L and *Cox*1 genes consistently grouped *G. firma* and *G. changii* samples into a single clade. Additionally, detailed comparisons of spermatangial types indicated that the “V”‐shaped and “P”‐shaped permatangia are actually the same type. This view has gained support from multiple studies (Abbott et al. 1991; Ng et al. 2017). However, because the samples analyzed by Ng et al. (2017) did not include specimens from the type locality or sequences compared directly to type specimens, their conclusion has not been widely accepted internationally. The Algaebase database also does not endorse this viewpoint (Guiry 2024). Furthermore, another species, *G. hainanensis* , has recently been added to the taxonomic debate surrounding *G. firma* and *G. changii*.

Wang et al. (2023) conducted an extensive diversity survey of Chinese Gracilariaceae. They obtained sequences of type and historical specimens and collected a large number of samples from type localities. Their samples of *G*.‘*firma*’ which they collected from the type locality of *G. firma* (Xindi, Xuwen Xian), shared sequence similarity with the type specimen of *G. hainanensis* , suggesting that *G. firma* may be synonymous with *G. hainanensis* , while *G. changii* represents a distinct entity. Their research highlights the importance of comparing historical specimens and sampling from type localities in Gracilariaceae taxonomy, and it contributed significantly to the development of a more comprehensive DNA sequence database. However, their study had certain limitations. First, they lacked a morphological comparison of *G. firma* and *G. hainanensis* , and therefore failed to consider differences in thallus size, color, and rhizoid structures. In our study, the average thallus length of *G. hainanensis* was 20–50 cm and it had dark red coloration and prominent rhizoids; these traits were consistent with the original description of *G. hainanensis* . Moreover, these features were absent in descriptions of *G. firma* provided by Li et al. (2023) and Xia and Zhang (1999). Second, Wang et al. (2023) did not compare their sequences with more GenBank entries. In the current study, phylogenetic trees based on *rbc*L and *Cox*1 genes revealed that sequences of historical specimens and newly collected samples of *G. changii* obtained by Wang et al. (2023) were highly similar to *G. firma* sequences collected by Ng et al. (2017), supporting the view that *G. firma* and *G. changii* are the same species. Additionally, sequences of *G. hainanensis* samples collected in our study clustered in the same clade as sequences of *G. ‘firma’* nd *G. hainanensis* obtained by Wang et al. (2023), clearly separating them from *G. firma/G. changii* sequences. In conclusion, the results of our study support the view of Ng et al. (2017) that *G. firma* and *G. changii* should be regarded as the same species, while *G. hainanensis* represents a distinct entity.

### 4.3 Seasonal and Ecosystem Variations in Gracilariaceae Diversity in Zhanjiang

Globally, 243 species and varieties of Gracilariaceae have been recorded (Guiry 2024), with most found in tropical waters. In this study, we identified eight Gracilariaceae species along the coast of Zhanjiang, Guangdong Province. They represent 25% of China's recorded species and 3.3% of the global total, which underscored the region's ecological significance. High species diversity was observed in HW and WST, where four and five species were recorded, respectively, and they exhibited extensive coverage across high, mid, and low tidal zones.

Significant differences in the diversity and coverage of Gracilariaceae species across different ecosystems were found (*p* < 0.05), with varying degrees of seasonal influence. In WST and EMG, the diversity and coverage of Gracilariaceae species were significantly higher in winter and spring compared to summer and autumn (*p* < 0.05), indicating strong seasonal effects. Similar patterns were observed in other intertidal zones with different substrates (unpublished data), aligning with Kam and Ang (2016), Zhang et al. (2021), and Liu et al. (2023), who reported comparable seasonal variations in macroalgal communities in adjacent intertidal zones. In contrast, macroalgae in northern China typically reach their growth peak in summer (Han and Liu 2014). However, in HW, TCI, and WSS, the diversity and coverage of Gracilariaceae species exhibited no significant variation across seasons (*p* > 0.05), highlighting the stability of these ecosystems.

The distribution and coverage of Gracilariaceae species are influenced by multiple environmental factors, such as temperature, light, and salinity, as well as by human activities and natural disturbances (Xu et al. 2009; Mendes et al. 2012; Mendoza‐Segura et al. 2023). Compared to the more stable environments of mangroves, seagrass beds, and saltwater ponds, tide pools and edges of mangroves, and other intertidal areas are more exposed and experience greater fluctuations in external factors such as tidal currents, water temperature, and salinity (Weitzman et al. 2021; Li et al. 2022). In summer, increased water temperature, light intensity, and longer daylight hours in the South China Sea likely accelerate tidal pool evaporation, raising salinity. Additionally, frequent rainfall during the summer rainy season causes dramatic fluctuations in the salinity of these open water bodies (Tang et al. 2022; Chen et al. 2023; Gao et al. 2023). These combined factors may collectively contribute to the observed decline in species diversity and coverage in the WST and EMG open ecosystems during summer. Moreover, from June to November, the region experiences peak typhoon activity, with intense hydrodynamic forces, rapid changes in water temperature and salinity, and potential habitat destruction, which may significantly affect the diversity and coverage of Gracilariaceae species (Jiang et al. 2020; Li et al. 2021).

In contrast, the enclosed environments of HW, TCI, and WSS are more stable, leading to reduced seasonal fluctuations in the diversity and coverage of Gracilariaceae species. The extensive root and leaf structures of seagrass in seagrass beds buffer water dynamics and create a stable microenvironment, thereby mitigating external environmental fluctuations (Han and Liu 2014; do Amaral Camara Lima et al. 2023). Seagrass beds are rich in organic matter and consistently supply nutrients, ensuring stable growth of Gracilariaceae species and minimizing seasonal variations (Miyajima and Hamaguchi 2019; Rahayu et al. 2019). In TCI, the canopy of mangrove trees reduces direct sunlight, while the root systems slow down water flow and trap nutrients, creating a relatively stable microhabitat (Srikanth et al. 2016; Revathy and Lakshmi 2024; Weaver and Stehno 2024). Consequently, Gracilariaceae species in TCI experience reduced impacts from seasonal disturbances. Similarly, WSS is a relatively closed system that exhibits stable salinity and nutrient availability, thereby sustaining consistent growth of Gracilariaceae species throughout the year (Salter 2018; Zhang et al. 2024).

### 4.4 Potential Species for Large‐Scale Cultivation in the South China Sea

Several studies have demonstrated that species with abundant natural resources and broad distribution often exhibit greater ecological adaptability, making them more suitable for artificial cultivation (Bhushan et al. 2023; Correia and Lopes 2023; Prazukin et al. 2024). *Gp. heteroclada*, the only *Gracilariopsis* species identified in this survey, exhibited wide distribution, a year‐round presence, and high coverage across multiple ecosystems. Pondevida and Hurtado‐Ponce (1996) reported similar observations in the Philippines. Luhan (1992), Kapraun et al. (1996), and Hurtado‐Ponce and Pondevida (1997) highlighted its superior agar gel strength (510–905 g cm^−2^), which is a characteristic not shared by the similarly widespread *G. salicornia* (200–290 g cm^−2^; Oyieke 1993; Buriyo and Kivaisi 2003; Lee et al. 2014). Additionally, *Gp. heteroclada* has already been established as a primary feed for abalone in the Philippines, and it has significant potential in food production, fertilizers, additives, and aquaculture (Capinpin Jr and Corre 1996; Chapman 2012; Guanzon Jr. et al. 2004). Our previous research demonstrated that *Gp. heteroclada* thrives at temperatures ranging from 25°C to 35°C (Huang et al. 2023), making it well suited to the warmer waters of the South China Sea. These findings underscore its strong potential for large‐scale cultivation in southern China.

*G. fisheri* and *G. edulis* were exclusively distributed in the tidal pools of Wushi, and they share similar morphological and ecological characteristics. Our research revealed that *G. fisheri* seedlings (< 0.5 mm) can enter a dormant state under unfavorable conditions and resume rapid growth when conditions improve, a critical feature for controlled cultivation, while carpospores form visible thalli in 4 weeks and reach 0.13–0.79 cm by week 9 under optimal conditions (Yang et al. 2025). Both species have been extensively studied internationally. In addition to their excellent agar gel strength and use as abalone feed (Chirapart et al. 2006; Meena et al. 2008), they have applications in pharmaceuticals, agriculture, and environmental remediation (Chaisuksant 2003; Kanjana et al. 2011; Singh et al. 2016; Gowdhami et al. 2019; Bhushan et al. 2023). Currently, *G. fisheri* is commercially cultivated in several Southeast Asian countries (Praiboon et al. 2006), while *G. edulis* has been successfully farmed on a large scale in India (Bhushan et al. 2023), generating significant economic benefits. Given their broad application potential and proven success in commercial cultivation in multiple countries, *G. fisheri* and *G. edulis* also hold substantial potential for large‐scale cultivation in southern China.

In this study, *G. hainanensis* was the only Gracilariaceae species identified in the mangrove ecosystem, where it maintained high and stable coverage (20%–23%) throughout all seasons. Previous research has shown that *Gracilaria* species in mangroves often exhibit higher agar content and gel strength compared to those in other ecosystems (Phang et al. 1996; Lee et al. 2016). The Chinese government places significant emphasis on the protection and development of mangrove ecosystems, and small‐scale ecological aquaculture has been initiated in these areas (Peng et al. 2013; Tengku Hashim et al. 2021). Given the good compatibility of *G. hainanensis* with mangrove ecosystems, it could be a suitable candidate for ecological polyculture in mangrove regions. However, *G. hainanensis* has been reported only in China and Vietnam (Xia and Zhang 1999; Guiry 2024), and its limited distribution has resulted in a severe lack of research on this species. To fully develop its cultivation potential, the primary task is to enhance fundamental research on *G. hainanensis* .

## 5 Conclusion

In this study, we systematically investigated the diversity, taxonomy, and ecological distribution of Gracilariaceae species across distinct ecosystems in Zhanjiang, China. Eight species were identified, including the newly recorded *G. fisheri* , and taxonomic ambiguities were resolved, confirming the synonymy of *G. firma* and *G. changii*. Diversity analysis revealed significant ecosystem‐specific and seasonal patterns, with tidal pools exhibiting higher diversity and seasonal variability, while enclosed ecosystems, such as mangroves and saltwater ponds, maintained lower diversity but greater stability. Based on the survey results and existing literature, we propose *Gp. heteroclada*, *G. fisheri* , *G. edulis* , and *G. hainanensis* as potential candidates for large‐scale cultivation in southern China due to their ecological adaptability, stable occurrence, and documented applications in agar production and aquaculture. This study supported the sustainable management and industrial development of Gracilariaceae resources in the South China Sea region.

## Author Contributions

**Zhaojun Zeng:** data curation (lead), formal analysis (lead), investigation (lead), methodology (lead), software (lead), visualization (lead), writing – original draft (lead). **Enyi Xie:** investigation (lead), funding acquisition (supporting), methodology (supporting). **Huaqiang Tan:** investigation (lead). **Xuefeng Wang:** investigation (lead). **Wencheng Yang:** investigation (lead), methodology (supporting). **Nenghui Li:** investigation (lead), methodology (supporting). **Qun Lai:** methodology (supporting), writing – original draft (supporting). **Kun Lin:** investigation (supporting). **Manning Lei:** investigation (supporting). **Xinlu Wu:** investigation (supporting). **Jianjun Cui:** conceptualization (lead), funding acquisition (lead), investigation (lead), methodology (equal), supervision (lead), writing – review and editing (lead).

## Ethics Statement

The authors have nothing to report.

## Conflicts of Interest

The authors declare no conflicts of interest.

## Supporting information


Table S1.

Table S3.

## Data Availability

The *rbc*L and *Cox*1 sequence data generated from this study are available publicly at https://www.ncbi.nlm.nih.gov under the GenBank accession numbers PQ818019 to PQ818043. The raw data for species coverage surveys are available in Table S3.
