# Supplementary material for: Diversity of Gracilariaceae (Gracilariales, Rhodophyta) Across Distinct Ecosystems in Zhanjiang, China: A Foundation for Screening Potential Cultivable Species in Southern China
Source: Ecol Evol. 2025 Jul 9;15(7):e71748. doi: 10.1002/ece3.71748 (PMC12240597; doi:10.1002/ece3.71748)
Supplement: Supplementary file 1 — Table S1. [file ECE3-15-e71748-s001.docx]

**TABLE S1** | Sample information for *rbcL* sequences from GenBank used in this study

| **Taxon** | **Collection Locality** | **Collection Date** | **Accession NO. of *rbcL*** | **Reference** |
| --- | --- | --- | --- | --- |
| *Gracilaria ‘firma’* | China | / | OP669599.1 | Wang et al. (2023) |
| *Gracilaria hainanensis* | China | / | OP669601.1 | Wang et al. (2023) |
| *Gracilaria arcuata* | / | / | AY049383.1 | Gurgel et al. (2004) |
| *Gracilaria salicornia* | OBS Camp 1, Ubin Island, Singapore | 26-Oct-2012 | KF831118.1 | Poh-Kheng et al. (2015) |
| *Gracilaria blodgettii* | China | / | OP669557.1 | Wang et al. (2023) |
| *Gracilaria coronopifolia* | USA | 18-Mar-2007 | KJ775800.1 | Conklin et al. (2014) |
| *Gracilaria lacinulata* | Playa Barranquita, Venezuela | / | AY049344.1 | Gurgel et al. (2004) |
| *Gracilaria tikvahiae* | Canada | / | AY049434.1 | Gurgel et al. (2004) |
| *Gracilaria incurvata* | Misaki, Japan | / | JQ026024.1 | Unpublished |
| *Gracilaria longa* | Napoli, Rivafiorita, Italy | / | AY651050.1 | Unpublished |
| *Gracilaria taiwanensis* | Taiwan, China | 09-Mar-2008 | HQ896848.1 | Lin et al. (2012) |
| *Gracilaria ornata* | Panama | / | AY049318.1 | Gurgel et al. (2004) |
| *Gracilaria textorii* | Japan | / | AY049325.1 | Gurgel et al. (2004) |
| *Gracilaria punctata* | Lungkeng, Kenting National Park, Taiwan, China | / | AY737446.1 | Unpublished |
| *Gracilaria firma* | Thi Nai Lagoon, Qui Nhon, Qinh Dinh, Viet Nam | 01-Apr-2005 | KY315293.1 | Ng et al. (2017) |
| *Gracilaria firma* | China | / | PP579586.1 | Li et al. (2023) |
| *Gracilaria changii* | China | / | OP669568.1 | Wang et al. (2023) |
| *Gracilaria* sp.2 | China | / | OP669704.1 | Wang et al. (2023) |
| *Hydropuntia* sp. | Malaysia |  | AB859153.1 | Unpublished |
| *Gracilaria edulis* | China | / | OP669574.1 | Wang et al. (2023) |
| *Gracilaria rangiferina* | Ghana | / | AY049379.1 | Gurgel et al. (2004) |
| *Gracilaria vermiculophylla* | Chiba, Inuwaka, Japan | 31-Jul-2004 | EF434912.1 | Yang et al. (2008) |
| *Gracilaria chilensis* | Chile | / | AY049396.1 | Unpublished |
| *Gracilaria tenuistipitata* | Dinh Vu, Viet Nam | 19-Apr-2003 | MH760425.1 | Gurgel et al. (2018) |
| *Gracilariopsis heteroclada* | China | / | OP669716.1 | Wang et al. (2023) |
| *Gracilariopsis lemaneiformis* | Chorrillos, Lima, Peru | 30-Dec-2014 | KP857577.1 | Arakaki et al. (2015) |
| *Gracilariopsis tenuifrons* | Itanhaem, Cibratel, Brazil | 17-Jun-2018 | OP903999.1 | Faria et al. (2023) |
| *Rhodymenia pseudopalmata* | North Carolina, Pender County, Topsail, USA | 05-Oct-2013 | KJ202094.1 | Unpublished |
| “/” stands for data not available. | | | | |

**TABLE S2** | Sample information for *cox1* sequences from GenBank used in this study

| **Taxon** | **Collection Locality** | **Collection Date** | **Accession NO. of *rbcL*** | **Reference** |
| --- | --- | --- | --- | --- |
| *Gracilaria* sp.2 | China | / | OP669514.1 | Wang et al. (2023) |
| *Gracilaria fisheri* | Magsaysay, R.T. Lim, Zamboanga, Sibugay, Philippines | 26-Aug-2016 | KY995690.1 | Unpublished |
| *Gracilaria fisheri* | Thailand | / | KY995691.1 | Unpublished |
| *Gracilaria changii* | China | / | OP669392.1 | Wang et al. (2023) |
| *Gracilaria firma* | Thi Nai Lagoon, Qui Nhon, Qinh Dinh, Viet Nam | 01-Apr-2005 | KY315253.1 | Ng et al. (2017) |
| *Gracilaria firma* | China | / | PP579577.1 | Li et al. (2023) |
| *Gracilaria punctata* | Kagoshima, offshore of Mageshima Island, Japan | 2016-05-18 | LC589307.1 | Suzuki et al. (2021) |
| *Gracilaria ferox* | Sao Paulo, Ubatuba, Praia Dura, Brazil | 13-Sep-2008 | JQ843330.1 | Costa et al. (2012) |
| *Gracilaria occidentalis* | Bermuda | / | KF761653.1 | Schneider et al. (2014) |
| *Gracilaria textorii* | China | / | KC782858.1 | Unpublished |
| *Gracilaria tikvahiae* | Rhode Island, USA | 01-Aug-2007 | FJ499547.1 | Saunders (2009) |
| *Gracilaria cervicornis* | Bahia, Entre Rios, Subauma, Brazil | 25-Oct-2010 | KP210156.1 | Lyra et al. (2015) |
| *Gracilaria incurvata* | Jongdal, Jeju, South Korea | 04-Apr-2010 | HQ322060.1 | Kim et al. (2010) |
| *Gracilaria taiwanensis* | China | / | OP669462.1 | Wang et al. (2023) |
| *Gracilaria dotyi* | / | / | HQ422938.1 | Sherwood et al. (2010) |
| *Gracilaria coronopifolia* | / | / | HQ423010.1 | Sherwood et al. (2010) |
| *Gracilaria arcuata* | China | / | OP669388.1 | Wang et al. (2023) |
| *Gracilaria pacifica* | USA | 23-May-2010 | HQ544260.1 | Unpublished |
| *Gracilaria gracilis* | Roscoff, France | 09-Apr-2005 | EF434919.1 | Yang et al. (2008) |
| *Gracilaria blodgettii* | China | / | OP669390.1 | Wang et al. (2023) |
| *Gracilaria hainanensis* | China | / | OP669436.1 | Wang et al. (2023) |
| *Gracilaria ‘firma’* | China | / | OP669433.1 | Wang et al. (2023) |
| *Gracilaria chilensis* | Caldera, Atacama, Chile | / | MN145983.1 | Unpublished |
| *Gracilaria tenuistipitata* | China | / | PP579573.1 | Li et al. (2023) |
| *Gracilaria salicornia* | Sinakan, Sabtang, Batanes, Philippines | 14-Apr-2016 | KY774622.1 | Unpublished |
| *Gracilaria vermiculophylla* | Asamushi, Japan | 26-Apr-2005 | HQ322046.1 | Kim et al. (2010) |
| *Gracilaria preissiana* | Australia | / | JQ026095.1 | Unpublished |
| *Gracilaria edulis* | China | / | PP579516.1 | Li et al. (2023) |
| *Gracilaria rangiferina* | Bahia, Santa Cruz de Cabralia, Apua, Brazil | 05-Nov-2010 | KP210186.1 | Lyra et al. (2015) |
| *Gracilariopsis lemaneiformis* | China | / | OP669551.1 | Wang et al. (2023) |
| *Gracilariopsis tenuifrons* | Rio de Janeiro, Cabo Frio, Brazil | 09-Nov-1987 | MH396181.1 | Iha et al. (2018) |
| *Gracilariopsis longissima* | United Kingdom | / | JQ843345.1 | Costa et al. (2012) |
| *Gracilariopsis heteroclada* | China | / | PP579596.1 | Li et al. (2023) |
| *Rhodymenia pseudopalmata* | North Carolina, Pender County, Topsail, USA | 05-Oct-2013 | KJ202084.1 | Unpublished |
| “/” stands for data not available. | | | | |

**References**

Wang X, Guo M, Yan S et al. 2023. Diversity of Gracilariaceae (Rhodophyta) in China: An integrative morphological and molecular assessment including a description of *Gracilaria tsengii* sp. nov. *Algal Research*, *71*, 103074.

Poh-Kheng N, Eem L P, Siew-Moi P. 2015. The first report of the parasitic red alga *Gracilaria babae* (Rhodophyta: Gracilariaceae) from Singapore. *Raffles Bulletin of Zoology*.

Conklin K Y, O'Doherty D C, Sherwood A R. 2014. *Hydropuntia perplexa*, n. comb.(Gracilariaceae, Rhodophyta), First Record of the Genus in Hawai ‘i1. *Pacific Science*, *68*(3), 421-434.

Gurgel C F D, Fredericq S. 2004. Systematics of the gracilariaceae (gracilariales, rhodophyta): a critical assessment based on *rbcl* sequence analyses 1. *Journal of phycology*, *40*(1), 138-159.

Lin S M, Liu L C, Payri C. 2012. Characterization of *Gracilaria vieillardii* (Gracilariaceae, Rhodophyta) and molecular phylogeny of foliose species from the western Pacific Ocean, including a description of *G. taiwanensis* sp. nov. *Phycologia*, *51*(4), 421-431.

Ng P K, Lin S M, Lim P E et al. 2017. Genetic and morphological analyses of *Gracilaria firma* and *G. changii* (Gracilariaceae, Rhodophyta), the commercially important agarophytes in western Pacific. *PLoS One*, *12*(7), e0182176.

Li N, Huang Q, Li H et al. 2023. Taxonomic study of four species of *Gracilaria* (Gracilariaceae, Rhodophyta) in Zhanjiang based on morphological and molecular data. *Journal of Tropical Oceanography*, 0. (in Chinese)

Yang E C, Kim M S, Geraldino P J L et al. 2008. Mitochondrial cox 1 and plastid rbc L genes of *Gracilaria vermiculophylla* (Gracilariaceae, Rhodophyta). Journal of applied phycology, 20, 161-168.

Gurgel C F D, Norris J N, Schmidt W E et al. 2018. Systematics of the Gracilariales (Rhodophyta) including new subfamilies, tribes, subgenera, and two new genera, *Agarophyton* gen. nov. and *Crassa* gen. nov. *Phytotaxa*, *374*(1), 1-23.

Arakaki N, Schmidt W E, Carbajal P et al. 2015. First occurrence of *Gracilaria chilensis*, and distribution of *Gracilariopsis lemaneiformis* (Gracilariaceae, Gracilariales) in Peru on the basis of *rbc*L sequence analysis. *Phytotaxa*, *208*(2), 175-181.

Faria A V, Martins N T, Ayres‐Ostrock L M et al. 2023. Phylogeography of the red alga *Gracilariopsis tenuifrons* (Gracilariales) along the Brazilian coast. *Journal of Phycology*, *59*(5), 1041-1052.

Suzuki M, Terada R. 2021. A new flattened species of *Gracilariopsis* (Gracilariales, Rhodophyta) from Japan. *Phycologia*, *60*(2), 158-163.

Costa E S, Plastino E M, Petti R et al. 2012. The gracilariaceae germplasm bank of the University of São Paulo, Brazil—a DNA barcoding approach. *Journal of Applied Phycology*, *24*, 1643-1653.

Schneider C W, Popolizio T R, Lane C E. 2014. Notes on the marine algae of the Bermudas. 14. Five additions to the benthic flora, including a distinctive second new species of *Crassitegula* (Rhodophyta, Sebdeniales) from the western Atlantic Ocean. *Phycologia*, *53*(2), 117-126.

Lyra G D M, Costa E D S, de Jesus P B et al. 2015. Phylogeny of Gracilariaceae (Rhodophyta): evidence from plastid and mitochondrial nucleotide sequences. *Journal of Phycology*, *51*(2), 356-366.

Saunders G W. 2009. Routine DNA barcoding of Canadian Gracilariales (Rhodophyta) reveals the invasive species *Gracilaria vermiculophylla* in British Columbia. *Molecular ecology resources*, *9*, 140-150.

Kim M S, Yang M Y, Cho G Y. 2010. Applying DNA barcoding to Korean Gracilariaceae (Rhodophyta). *Cryptogamie Algologie*, *31*(4), 387.

Sherwood A R, Kurihara A, Conklin K Y et al. 2010. The Hawaiian Rhodophyta Biodiversity Survey (2006-2010): a summary of principal findings. *BMC plant biology*, *10*, 1-29.

Iha C, Grassa C J, Lyra G D M et al. 2018. Organellar genomics: a useful tool to study evolutionary relationships and molecular evolution in Gracilariaceae (Rhodophyta). *Journal of Phycology*, *54*(6), 775-787.
